# Significant increased CA199 levels in acute pancreatitis patients predicts the presence of pancreatic cancer

**DOI:** 10.18632/oncotarget.23993

**Published:** 2018-01-04

**Authors:** Dongling Teng, Keyan Wu, Yunyun Sun, Min Zhang, Dan Wang, Jian Wu, Tao Yin, Weijuan Gong, Yanbing Ding, Weiming Xiao, Guotao Lu, Weiqin Li

**Affiliations:** ^1^ Department of Gastroenterology, Affiliated Hospital of Yangzhou University, Yangzhou University, Yangzhou 225000, Jiangsu, China; ^2^ Laboratory of Gastroenterology, Affiliated Hospital of Yangzhou University, Yangzhou University, Yangzhou 225000, Jiangsu, China; ^3^ Surgical Intensive Care Unit (SICU), Department of General Surgery, Jinling Hospital, Medical School of Nanjing University, Nanjing 210002, Jiangsu, China

**Keywords:** acute pancreatitis, CA199, pancreatic cancer, retrospective study

## Abstract

**Background and study aims:**

Carbohydrate antigen 19-9 (CA199) has been identified as a tumor marker for pancreatic cancer but also increases in benign lesions of the digestive system. However, literature associated with the relationship between CA199 and acute pancreatitis (AP) is limited. This study aimed to focus on serum CA199 level measurements in AP patients and the associated clinical significance.

**Materials and methods:**

From January 2006 to December 2015, 1,609 consecutive patients with AP were admitted to our department and included in the study. The relationships among the etiology of AP, the disease severity, the incidence of pancreatic cancer during hospitalization and CA199 levels were analyzed.

**Results:**

Serum CA199 levels were measured for 693 of 1,609 AP patients. Of those patients, 186 (26.8%) had elevated CA199 levels (> 37 U/ml). Patients with high CA199 levels were older and had predominantly biliary causes in comparison with patients with normal CA199 levels. There were no definite specific correlations between CA199 levels and disease severity in AP. In addition, serum levels of CA199 positively correlated with serum alanine aminotransferase, aspartate transaminase, glutamyl transpeptidase, alkaline phosphatase and creatinine levels. After stratification, the incidence of pancreatic cancer increased proportionally to CA199 levels in AP patients.

**Conclusions:**

Serum CA199 levels was elevated in patients with AP, especially in patients with biliary pancreatitis. AP patients with significantly increased CA199 levels may have a higher risk for the presence of pancreatic cancer. We recommended routinely monitoring CA199 levels during hospitalization for AP patients.

## INTRODUCTION

Acute pancreatitis (AP) is characterized by pancreatic tissue self-digestion, edema, hemorrhage and even necrosis caused by the inflammatory responses next to trypsin activation. The signs and symptoms of AP include pain in the upper abdomen, nausea, vomiting, fever and increased serum amylase levels. The revised Atlanta classification categorizes AP into mild, moderate, and severe categories based upon different disease severities [1].

AP has multiple etiologies [2, 3], and the most common causes include gallstones, heavy alcohol consumption and hypertriglyceridemia. Other minor causes include pancreatic duct obstruction, surgery and trauma, endocrine and metabolic disorders, infections, drugs, genetic factors and so on. In addition, 5–25% of AP cases are idiopathic with an unknown etiology [4, 5]. It was recently reported that AP may be an early manifestation of pancreatic cancer [6–8]. Hence, many pancreatic cancer patients may be misdiagnosed as AP, even delaying their diagnosis and treatment [9, 10].

Carbohydrate antigen 19-9 (CA199) is a glycoprotein macromolecule that is first discovered by Koprowski in 1979 [11] and has been used as a tumor marker for pancreatic cancer (PC) since 1983 [12, 13]. CA199 is still considered to be the most sensitive serum marker for pancreatic cancer [14]. Recently, some research reported that CA199 could also be elevated in other digestive system tumors and in patients with benign hepatobiliary [15–17] and gastrointestinal disease [18, 19]. However, the relationship between CA199 levels in AP patients and the presence of pancreatic cancer remains less observed and has not been investigated thoroughly.

In this study, patients with a definite diagnosis of AP admitted to our hospital over a period of 10 years were retrospectively collected. We aimed to analyze the comparisons in CA199 levels in different AP groups, and to determine whether elevated CA199 levels in AP patients was associated with the risk factors for pancreatic cancer.

## RESULTS

### AP patient demographics and baseline characteristics

A total of 1,609 consecutive AP patients from January 2006 to December 2015 were reviewed for this study, including 903 males and 706 females, with an average age of 54.8 years. The most common cause of AP was biliary (*N* = 606, 37.7%), followed by hypertriglyceridemia (*N* = 300, 18.7%) and alcohol consumption (*N* = 150, 9.3%), and other causes accounted for 34.3% of cases. Among these 1,609 AP patients, 1,185 (73.6%) had MAP, 342 (21.3%) had MSAP, and 82 (5.1%) had SAP.

### CA199 levels and baseline characteristics

**Figure 1:**
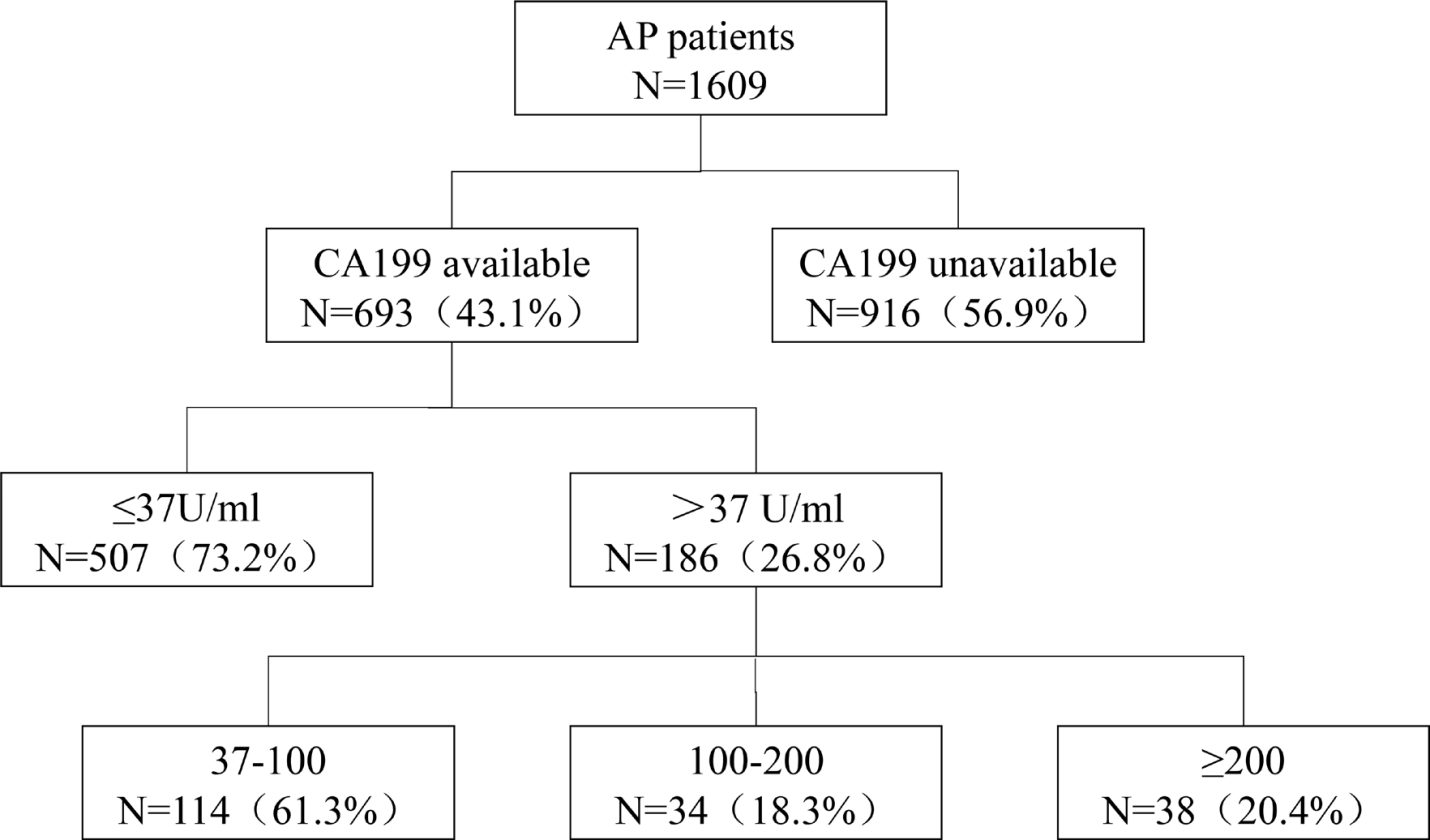
The distribution of CA199 in patients with greater than 37 U / ml levels

Serum CA199 levels were measured for 693 of 1,609 AP patients during hospitalization (Figure 1). Of those patients, 404 (58.3%) were male, and the average age was 56.4 years. Biliary was the most common AP etiology (38.5%), followed by hypertriglyceridemia (20.1%) and alcohol consumption (9.5%), and other causes accounted for 31.9% of cases. In these cases, 510 (73.6%) had MAP, 152 (21.9%) had MSAP, and 31 (4.5%) had SAP.

Among these 693 AP patients, 507 (73.2%) had normal serum CA199 levels and 186 (26.8%) had elevated CA199 levels. These 186 patients with high CA199 levels were older (average age 62.7 vs. 54.8 years, *P* < 0.001) and had higher percentage of biliary pancreatitis (53.8% vs. 32.9%, *P* < 0.001) than patients with normal CA199 levels. The data were presented in Table 1.

**Table 1:** Comparison of clinical characteristics and outcomes between AP patients with vs. without high serum CA199 levels

	All	High CA199s	NormalCA199s	
	*N* = 693	*N* = 186	*N* = 507	*P* value
Age (mean ± sd), yrs	56.4 ± 15.6	62.7 ± 14.3	54.8 ± 15.5	< 0.001
Male sex, N (%)	404 (58.3)	10 5 (56.5)	229 (45.2)	0.551
Underlying diseases, N (%)				
Diabetes	110 (15.9)	28 (15.1)	82 (16.2)	0.184
Coronary heart disease	32 (4.6)	11 (6.5)	21 (4.1)	0.745
hypertension	109 (15.7)	42 (4.6) 67 (13.2)		0.060
Etiology, N (%)				< 0.001
Biliary	267 (38.5)	100 (53.8)	167 (32.9)	
Alcohol	66 (9.5)	21 (11.3)	45 (8.9)	
Hypertriglyceri	139 (20.1)	14 (7.5)	125 (24.7)	
demia				
Others	221 (31.9)	51 (27.4)	170 (33.5)	
Severity, N (%)				0.539
MAP	510 (73.6)	140 (75.3)	371 (73.2)	
MSAP	164 (21.9)	36 (19.4)	115 (22.7)	
SAP	35 (4.5)	10 (5.4)	21 (4.1)	
Pancreatic cancer	10 (1.4)	8 (4.3)	2 (0.4)	0.001
Smoking, N (%)	142 (20.5)	25 (13.4)	117 (23.1)	0.005
Drinking, N (%)	103 (14.9)	20 (10.8)	83 (16.4)	0.065

Then, the patients with elevated CA199 levels were further divided into three groups according to serum CA199 levels: 37–100 (*N* = 114, 61.3%), 100–200 (*N* = 34, 18.3%), and > 200 (*N* = 38, 20.4%) (Table 2). Similarly, it was observed that patients with higher serum CA199 levels were more likely to be older (*P* < 0.001) and to have biliary pancreatitis (*P* < 0.001). The data were presented in Table 2.

### CA199 levels and clinical outcomes

**Figure 2:**
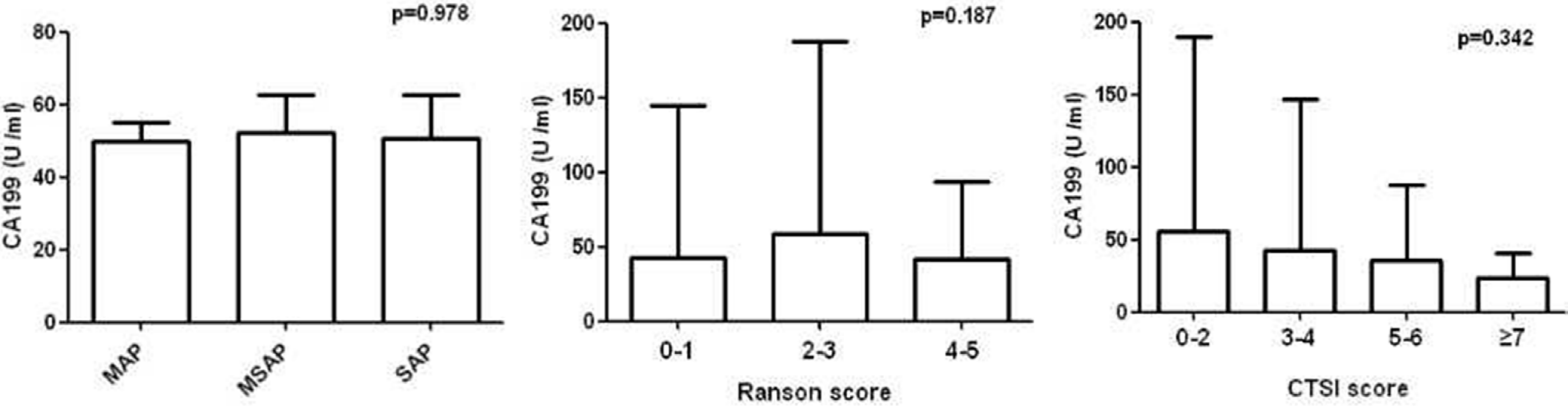
Comparison of serum CA199 concentrations by Atlanta classification, Ranson and CTSI score

As shown in Table 2 and Figure 2, there were no specific correlations between CA199 levels and AP disease severity. Moreover, Ranson score and CT severity index (CTSI) were used to assess the disease severity of AP [1], and we failed to find significant relationship between the serum CA199 levels and disease severity scoring (Ranson score and CTSI).

**Table 2:** Comparison of clinical characteristics and outcomes between different CA199 categories in patients with AP

	CA199 (U/ml)				*P* value
	≤37	37–100	100–200	> 200	
	(*n* = 507)	(*n* = 114)	(*n* = 34)	(*n* = 38)	
Age (mean ± sd), yrs	54.8 ± 15.5	60.6 ± 14.8	66.4 ± 13.1	65.5 ± 13.1	< 0.001
Male sex, N (%)	299 (45.2)	62 (54.4)	19 (55.9)	24 (63.2)	0.738
Underlying diseases, N (%)					
Diabetes	82 (16.2)	18 (15.8)	3 (8.8)	7 (18.4)	0.466
Coronary heart disease	21 (4.1)	6 (5.3)	3 (8.8)	2 (5.3)	0.828
hypertension	67 (13.2)	28 (24.6)	7 (20.6)	7 (18.4)	0.094
Etiology, N (%)					< 0.001
Biliary	167 (32.9)	59 (51.8)	19 (55.9)	22 (57.9)	
Alcohol	45 (8.9)	12 (10.5)	5 (14.7)	4 (10.5)	
Hypertriglyceridemia	125 (24.7)	12 (10.5)	0 (0)	2 (5.3)	
Others	170 (33.5)	31 (27.2)	10 (29.4)	10 (26.3)	
Severity, N (%)					0.78
MAP	371 (73.2)	89 (78.1)	24 (70.6)	27 (71.1)	
MSAP	115 (22.7)	20 (17.5)	7 (20.6)	9 (23.7)	
SAP	21 (4.1)	5 (4.4)	3 (8.8)	2 (5.3)	
Pancreatic cancer	1 (0.2)	0 (0)	2 (5.9)	6 (15.8)	< 0.001
Smoking, N (%)	117 (23.1)	13 (11.4)	4 (11.8)	8 (21.1)	0.024
Drinking, N (%)	83 (16.4)	12 (10.5)	3 (8.8)	5 (13.2)	0.299

**Figure 3:**
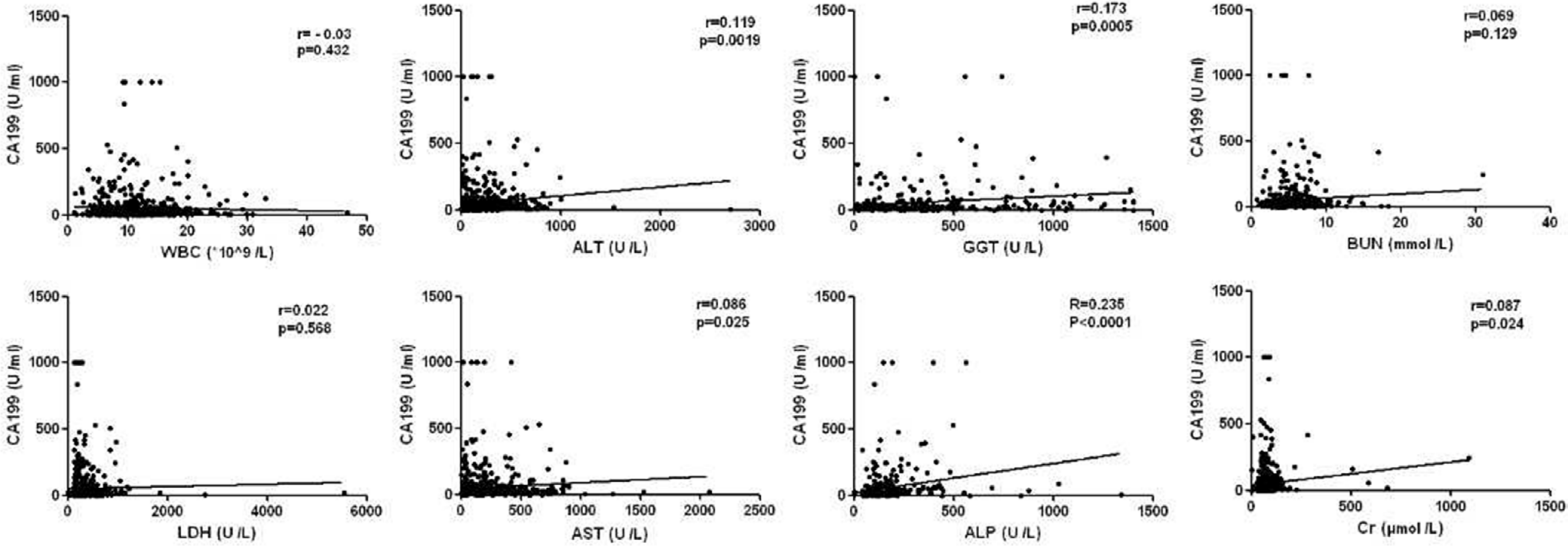
Correlation between CA199 and other clinical indicators in AP patients

In addition, the correlations between serum CA199 levels and the other clinical serological parameters of AP patients were analyzed. As shown in Figure 3, CA199 levels correlated positively with alanine aminotransferase (ALT), aspartate transaminase (AST), glutamyl transpeptidase (GGT), alkaline phosphatase (ALP) and creatinine (Cr) levels, with corresponding *r* values of 0.119 (*P* = 0.0019), 0.086 (*P* = 0.025), 0.173 (*P* = 0.0005), 0.235 (*P* < 0.0001) and 0.087 (*P* = 0.024) respectively, while no significant correlations with white blood cell (WBC), lactate dehydrogenase (LDH) and blood urea nitrogen (BUN) levels were observed (r = -0.03, 0.022, 0.069, respectively).

### CA199 levels and the presence of pancreatic cancer

**Figure 4:**
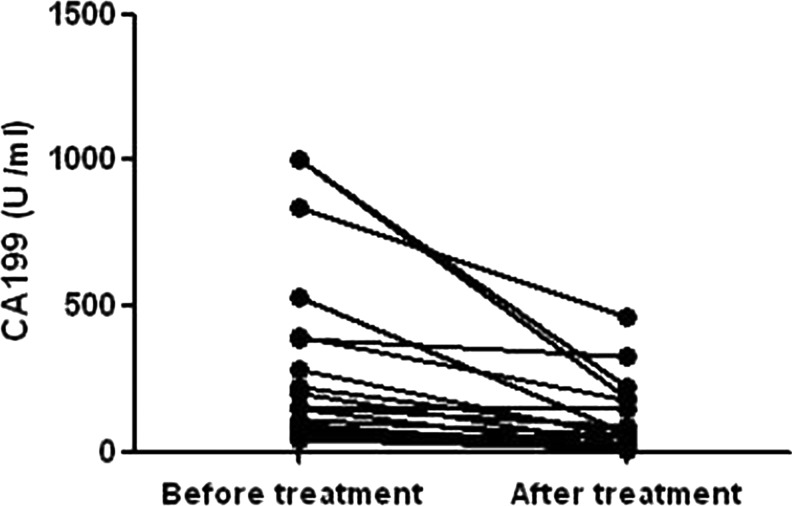
Dynamic changes of CA199 in AP patients

Of the 186 AP patients with elevated CA199 levels, 27 patients had their CA199 levels re-measured during hospitalization, and CA199 levels of 26 patients had decreased significantly (Figure 4). The elevation of re-measured CA199 levels was observed in one patient, and who turned out to have pancreatic cancer.

Furthermore, increased CA199 levels (CA199 > 37 U/ml) accounted for 26.8% of cases (186/693), of which 8 were associated with pancreatic cancer. The mean CA199 value in 8 patients with pancreatic cancer was 338.2 U/ml, and the mean CA199 value in non-pancreatic cancer patients was 137.5 U/ml. The incidence of pancreatic cancer tended to be much higher in AP patients with elevated serum CA199 levels than with normal CA199 levels, as shown in Table 1. Eight of the 186 AP patients with high CA199 levels suffered from pancreatic cancer, while only 2 of the 507 AP patients with normal serum CA199 levels had pancreatic cancer. In addition, pancreatic cancer was present in 2/507 (0.4%) of AP patients with normal CA199 levels, 0/114 (0%) of AP patients with CA199 levels of 37–100 U / ml, 2/34 (5.9%) with CA199 levels of 100–200 U / ml, and 6/38 (15.8%) with CA199 levels > 200 U / ml (*P* < 0.001, Table 2). The higher serum CA199 levels, the greater the risk for the presence of pancreatic cancer was observed in our study.

### Testing for selection bias

To validate for the absence of the selection bias in the AP patients included in this study, comparisons were performed between patients with available serum CA199 levels (*N* = 693) and patients with serum CA199 levels unavailable (*N* = 921). Demographics and clinical characteristics showed no significant differences between these two groups, except for age and personal history, as shown in Table 3. Patients with available CA199 levels were older (57.0 vs. 53.2 years, *P* < 0.001). No significant differences in disease severity and underlying diseases were identified between the two groups.

**Table 3:** Comparison of baseline clinical characteristics for patients with vs. without serum CA199 levels

	CA199 measured	CA199 not vailable	
	N = 693	N = 916	*P* value
Age (mean ± sd), yrs	57.0 ± 15.6	53.2 ± 17.1	< 0.001
Male sex, N (%)	404 (58.3)	499 (54.5)	0.126
Underlying diseases, N (%)			
Diabetes	110 (15.9)	115 (12.6)	0.057
Coronary heart disease	32 (4.6)	37 (4.0)	0.779
hypertension	109 (15.7)	133 (14.5)	0.502
Etiology, N (%)			0.314
Biliary	267 (38.5)	339 (37.0)	
Alcohol	66 (9.5)	84 (9.2)	
Hypertriglyceridemia	139 (20.1)	162 (17.7)	
Others	221 (31.9)	331 (36.1)	
Severity, N (%)			0.551
MAP	510 (73.6)	675 (73.7)	
MSAP	152 (21.9)	190 (20.7)	
SAP	31 (4.5)	51 (5.6)	
Smoking, N (%)	142 (20.5)	104 (11.4)	< 0.001
Drinking, N (%)	103 (14.9)	88 (9.6)	0.001

## DISCUSSION

CA199 is a polymer glycoprotein with a sialylated type I lactose fucoidan structure that consists of six glycosyl groups (sialylated Lewis A antigen) [22, 23]. Trace CA199 levels are present in healthy adult salivary glands and in prostate, pancreas, breast, stomach, bile duct, gallbladder, and bronchial epithelial cells [24]. Recent studies have shown that serum CA199 levels could be elevated in gastrointestinal tumors and in benign hepatobiliary and gastrointestinal disease, but there lacked research on CA199 expression in patients with AP. To our knowledge, our study is by far the largest clinical study demonstrating the significance of CA199 expression in patients with AP. Our study totally included 1,609 AP patients, of which 693 patients had serum CA199 levels measured during hospitalization and 186 patients had elevated CA199 levels (> 37 U/ml), with a positive rate of 26.8%.

Previous studies have shown that 46% of patients with choledocholithiasis had increased serum CA199 levels [25], and this rate was even higher when acute cholangitis was included. The reason for this phenomenon may lie in that inflammation stimulates the bile duct epithelial cells to over-secrete CA199 and increases the permeability of the bile duct wall. Furthermore, bile excretion obstruction induces the bile reflux, and then CA199 is secreted into the circulation.

In this clinical research, our data showed that the most common cause of AP was biliary, which was consistent with previous studies in China [26–28]. Additionally, the incidence of high serum CA199 levels was higher in patients with biliary pancreatitis than in patients with hypertriglyceridemic and alcoholic AP. Moreover, we found that serum CA199 levels correlated positively with ALT, AST, GGT and ALP levels, which may be associated with the phenomenon of the serum CA199 levels was higher in biliary pancreatitis patients. In addition, our study also found that CA199 levels were positively associated with the serum creatinine levels; this result may mainly cause by acute kidney injury and was consistent with previous reports [29, 30]. However, despite these above clinical findings, we failed to observe strong correlation between CA199 levels and disease severity (Atlanta disease grade, Ranson score and CTSI), which suggested that CA199 levels could not be used as an indicator of AP severity.

AP could be induced by pancreatic tumor mass which causes pancreatic duct obstruction and presents as the first clinical manifestation of pancreatic cancer, which accounts for approximately 2% of total AP cases [31, 32]. Due to the pancreatic edema and necrosis characteristics that are present in AP patients, it is difficult to distinguish tumor mass lesions through imaging examinations, which results in delayed diagnoses of pancreatic cancer [33]. Currently, there is no effective way to judge whether AP patients are complicated with pancreatic cancer at the same time. As a tumor-associated antigen, CA199 is commonly used to assess the diagnosis and treatment of pancreatic cancer [13]. Thus, we expect that increased CA199 levels can serve as an early warning sign for AP patients with pancreatic cancer. Surprisingly, we found an incidence of pancreatic cancer as high as 1.3% among AP patients. A significant increase in serum CA199 levels could suggest a risk of AP complicated with pancreatic cancer. Furthermore, when CA199 > 200 U/ml, the incidence of pancreatic cancer among AP patients was as high as 15.8%. More importantly, elevations in CA199 levels caused by benign lesions would decrease after pancreatic inflammatory resolution, in contrast, the CA199 levels in AP patients with pancreatic cancer remained unchanged or even higher. These results suggested that a significant increase in serum CA199 levels in patients with AP may be an effective and sensitive indicator of pancreatic cancer.

This study has several limitations. First, it is a single-center retrospective study. Second, the diagnosis of pancreatic tumor mass was based on clinical symptoms and imaging diagnosis and some patients lacked histopathological reports. Third, dynamic monitoring of CA199 changes was performed for limited patients. Prospective, multi-center studies are needed to validate the obtained findings and to further explore the clinical significance of CA199 as an early indicator of pancreatic cancer in AP patients in future.

In conclusion, our study confirmed that CA199 was elevated in AP patients and was more common in cases of biliary pancreatitis. CA199 levels in AP patients without pancreatic cancer decreased rapidly after AP recovery. Among AP patients with significantly increased CA199 levels, there was a positive correlation between CA199 levels and the incidence of pancreatic cancer. We strongly recommend that patients with AP should be routinely examined for tumor markers and should be actively followed up after discharge from the hospital to avoid missing pancreatic tumors.

## MATERIALS AND METHODS

### Patient selection

AP patients admitted to the Affiliated Hospital of Yangzhou University from January 2006 to December 2015 were included in this study. According to the 2012 revised Atlanta classification [1], diagnosis of AP requires at least 2 of the following criteria: acute abdominal pain, elevated serum amylase (more than three times the upper limit of the normal range), and an imaging study with characteristic changes (CT, MRI, abdominal ultrasound or endoscopic ultrasound). For patients who were re-hospitalized for several times during the study period, only the first admission was included. The exclusion criteria for the study were as follows: a. patients under 18 years old, b. pregnant women, c. endoscopic retrograde cholangiopancreatography (ERCP) induced pancreatitis, and d. AP caused by direct trauma.

Due to the retrospective characteristics of the study from 2006 to 2015, informed consent was waived and the study was approved by the Ethics Committees of our hospital.

### Severity of acute pancreatitis, assessment of complications and diagnosis of pancreatic cancer

According to the 2012 revised Atlanta classification [1], AP is divided into mild acute pancreatitis (MAP), moderate severe acute pancreatitis (MSAP) and severe acute pancreatitis (SAP). MAP is not associated with organ failure and local or systemic complications. MSAP is accompanied by transient organ failure and/or local or systemic complications within 48 hours, without persistent organ failure. SAP should be accompanied by persistent organ failure over more than 48 hours and can involve one or more organs. Local complications of AP include acute per-pancreatic fluid collection (APFC), pancreatic pseudocyst, acute necrotizing collection (ANC), and walled-off necrosis (WON). Systemic complications include systemic inflammatory response syndrome(SIRS), acute respiratory distress syndrome (ARDS), acute kidney injury (AKI), shock and others [20, 21].

The diagnosis of pancreatic cancer is primarily based on serum CA199 levels and pancreatic imaging examination, along with the histopathology findings of operative specimens. Enhanced CT or other tests may reveal a solid mass or diffuse enlargement of the pancreas, and sometimes along with an uneven low-density signal, tumor distal pancreatic atrophy and pancreatic duct dilatation, which indicates the diagnosis of pancreatic cancer.

### Detection of CA199 levels

CA199 levels were determined by using the Backman Dxi800 automatic chemiluminescence method. Venous blood samples of the fasting patients were collected in the morning, and the serum was separated within 2 hours. CA199 level > 37 U/mL was regarded as elevated while CA199 level < 37 U/mL was regarded as normal. In our hospital, the maximum range of CA199 detection was 1000 U/ml, therefore, the results were recorded as 1000 U/ml if it exceeds the range.

### Statistical analysis

As to the baseline characteristics, AP etiology, disease severity, personal history, and clinical outcomes were compared according to different CA199 levels. We then compared baseline characteristics and clinical outcomes between different CA199 levels categories (0–37, 37–100, 100–200, greater than 200). Statistical analysis was performed by using the version 16.0 SPSS software (IBM Analytics, Armonk, NY). Data are presented as mean ± standard deviation (SD) for continuous variables and absolute numbers and percentages for categorical variables. Student *t* test or Mann–Whitney test was used for analyzing continuous variables and the Chi-square test was used for analyzing categorical variables. Correlation analyses were performed by using Pearson’s simple correlation test. Patient characteristics were compared between patients with available serum CA199 levels and patients without to investigate for the selection bias in our study. A probability (*p* value) of < 0.05 was considered statistically significant.

## Abbreviations

AP: Acute pancreatitis
CA199: Carbohydrate antigen 199
PC: Pancreatic cancer
MAP: Mild acute pancreatitis
MSAP: Moderate severe acute pancreatitis
SAP: Severe acute pancreatitis
SIRS: Systemic inflammatory response
ARDS: Acute respiratory distress syndrome
CT: Computed tomography scan
CTSI: Computed tomography severity index
ALT: Alanine aminotransferase
AST: Aspartate transaminase
GGT: Glutamyl transpeptidase
ALP: Alkaline phosphatase
Cr: Creatinine
WBC: White blood cell
LDH: Lactate dehydrogenase
BUN: Blood urea nitrogen
